# Introgression of wild alleles into the tetraploid peanut crop to improve water use efficiency, earliness and yield

**DOI:** 10.1371/journal.pone.0198776

**Published:** 2018-06-11

**Authors:** Wellison F. Dutra, Yrlânia L. Guerra, Jean P. C. Ramos, Pedro D. Fernandes, Carliane R. C. Silva, David J. Bertioli, Soraya C. M. Leal-Bertioli, Roseane C. Santos

**Affiliations:** 1 Federal University of Paraíba, Agronomy Pos-Graduation, Rodovia PB 079, km 12, CEP, Areia, PB, Brazil; 2 State University of Paraíba, Pró-Reitoria de Pós-Graduação e Pesquisa, Rua Baraúnas, n° 351, Universitário, CEP, Campina Grande, PB, Brazil; 3 Laboratory of Biotechnology, Embrapa Cotton, Rua Osvaldo Cruz, n° 1143, Centenário, CEP, Campina Grande, PB, Brazil; 4 Crop and Soil Science Department/Center for Applied Genetic Technologies, University of Georgia, Athens, GA, United States of America; 5 Plant Pathology Department/Center for Applied Genetic Technologies, University of Georgia, Athens, GA, United States of America; Institute of Genetics and Developmental Biology Chinese Academy of Sciences, CHINA

## Abstract

The introduction of genes from wild species is a practice little adopted by breeders for the improvement of commercial crops, although it represents an excellent opportunity to enrich the genetic basis and create new cultivars. In peanut, this practice is being increasingly adopted. In this study we present results of introgression of wild alleles from the wild species *Arachis duranensis* and *A*. *batizocoi* improving photosynthetic traits and yield in a set of lines derived from the cross of an induced allotetraploid and cultivated peanut with selection under water stress. The assays were carried out in greenhouse and field focusing on physiological and agronomic traits. A multivariate model (UPGMA) was adopted in order to classify drought tolerant lines. Several lines showed improved levels of tolerance, with values similar to or greater than the tolerant control. Two BC_1_F_6_ lines (53 P4 and 96 P9) were highlighted for good drought-related traits, earliness and pod yield, having better phenotypic profile to the drought tolerant elite commercial cultivar BR1. These lines are good candidates for the creation of peanut cultivars suitable for production in semiarid environments.

## Introduction

Drought is a widespread environmental phenomenon with particularly damaging social and economic consequences in arid and semiarid environments. The development of plant cultivars adapted to environments prone to drought is a valuable strategy in improvement programs and a great challenge due to complex genetic inheritance [1]. Drought response involves cascades of events with consequences in biochemistry, physiology, and phenotype [2, 3]. To simplify the process of selection, breeders can use surrogate traits in order to assist the identification of plants tolerant to drought.

Plants under water stress have altered gas exchange due to diffusive limitations of CO_2_, which decreases carboxylation efficiency, or due to limitations of chloroplast activity caused by photo inhibition [2]. Several protective mechanisms have been developed by plants in order to balance absorbed light energy with photosynthesis. According to Kalariya et al. [4], non-photochemical quenching (NPQ) is a very important trait, which refers to non-photochemical releasing of excess energy through the chloroplasts, protecting the photosynthetic apparatus. Gas exchange and chlorophyll *a* fluorescence are very sensitive indicators of physiological status of leaves and plant performance in a wide range of situations [2, 5]. They reveal the current state of the photosynthetic metabolism, including the status of damage and repair under stress conditions [4, 5].

Peanut (*Arachis hypogaea* L.) is an important oilseed cultivated in many countries, to attend grain and oil markets. The genus *Arachis* has over 80, mostly diploid (2*n* = 2*x* = 20) species, which represent valuable genetic resources with wide adaptation to tropical and semiarid environments [6, 7]. The use of wild species of *Arachis* in improvement programs has been limited, mainly due ploidy differences and chromosomic barriers among the species. This can be overcome by artificial hybridizing A and B genome wild species followed by induced chromosome duplication to restore fertility and the tetraploid state [8]. The development of synthetic lines by combining A and B genomes, has provided a range of tetraploids possessing several good traits, such as resistance to diseases and insect pests, and opened new opportunities for peanut improvement [9–12]. Varieties such as ‘Tamnut 74’ [13], ‘Coan’ [14] and ‘NemaTAM’ [15], ‘Tifguard’ [16] and Bailey [17] that have a genetic contribution from wild *Arachis* species, were released for cultivation in the USA.

In Brazil, introgression efforts were initiated in 2000, by a multidisciplinary team of EMBRAPA (Brazilian Agricultural Research Corporation), in collaboration with other national and international institutions, focusing on obtaining synthetic allotetraploid lines resistant to foliar diseases. Currently, several synthetics allotetraploid are available and are being evaluated for drought tolerance [11, 18,19]. Robust commercial cultivars are being used as parents in crossbreeding work. Recently, three commercial cultivars were released in Senegal with improved disease resistance and yield [20].

In this work, we report the development of breeding lines derived from the cross between *A*. *hypogaea* subsp. *fastigiata* cv. BR1, widely grown in Brazilian semiarid region due to broad environmental adaptation [21], and the induced allotetraploid (*A*. *batizocoi* K9484 x *A*. *duranensis* SeSn 2848)^4x^ [11]. The parent *A*. *duranensis* SeSn 2848 was originally found in a semi-arid region of Argentina, and was found to have conservative transpiration behavior, that could be advantageous for introgression. Lines had improved drought-related traits, such as water use efficiency, high productivity, and early flowering, all desirable traits for cultivation in areas of low water availability.

## Material and methods

### Plant material

**Fig 1:**
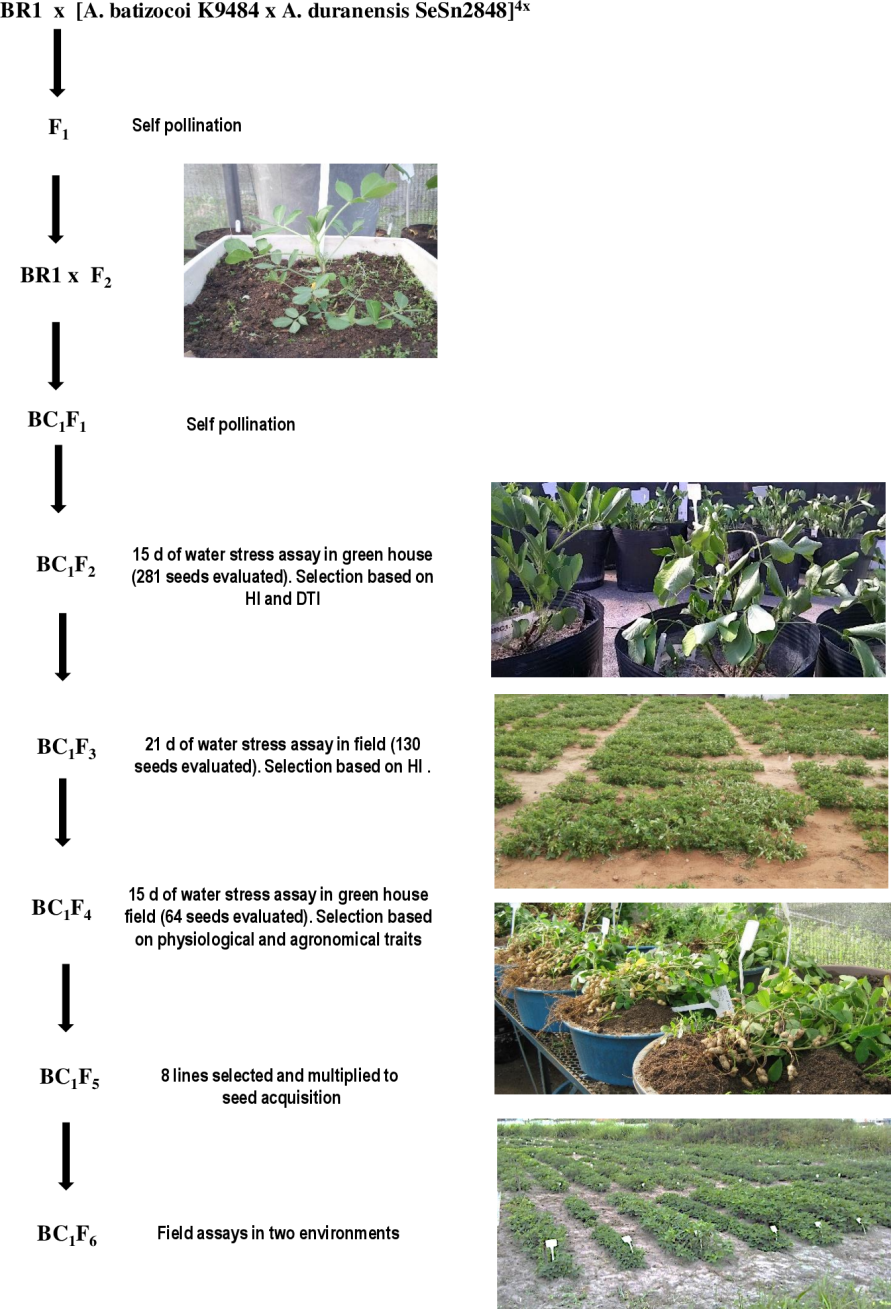
Summary of the selection steps adopted in breeding of induced allotetraploid BatDur.

*A*. *hypogaea* subsp. *fastigiata* var. *fastigiata* cv. BR1 (here called BR1) is an early-upright cultivar widely adapted to tropical and semiarid environments [3, 21]. It was chosen as a parent due to high ability to produce mature pods even with low water availability, both intermittent and end of season [22]. The induced allotetraploid [*A*. *batizocoi* K9484 x *A*. *duranensis* SeSn2848]^4x^ (here called BatDur), was produced using wild accessions from the *Arachis*-germplasm bank at EMBRAPA Genetic Resources and Biotechnology [11]. BR1 and BatDur were crossed and F_2_ progeny from this hybrid were backcrossed with BR1. BC_1_F_1_s were selfed, generating 281 seeds. BC_1_F_2_ plants were grown in green house (Recife, ‎8°03′14″S 34°52′51″W, 7m), seeds were sown in 20L pots containing sandy-loam textured soil previously limed and fertilized (NPK, 20:60:30, ammonium sulfate, single superphosphate and potassium chloride). Twenty-five days after germination, plants were submitted to water withdrawal for 15 days. Only 87 reached full cycle, and among them 13 were selected based on harvest index (HI ≥ 35%) and drought tolerance index (DTI ≥ 0.7) (S1 Table). HI was estimated based on pod yield/total plant dry weight ratio [23], and DTI was estimated through pod yield under stressed treatment/pod yield under control treatment ratio [24]. As all progenies were submitted to stress, the mean of BR1 was used as control. Ten BC_1_F_3_ seeds from each of the 13 selected plants were selected for further field assays. A summary of the selection steps of all procedures is found in Fig 1.

### Initial field selection and physiological assays in green house

One hundred and thirty BC_1_F_3_ seeds were grown in field trial (Campina Grande, PB, 7°13’50”S, 35°52’52”W, 551 m, semiarid climate), during end of rainy season 2015 (July-October). Plants were sown in 5m-rows, spaced 30 cm, and after 25 days of plant emergence submitted to 21 days of water withdrawal. Thereafter, the irrigation was restored, maintaining watering equivalent to 400 mm during the growing cycle [25]. Crop management was followed according to Santos et al. [26]. At harvest, 64 out of the initial 130 plants were selected based on harvest index (HI ≥ 30%) (S1 Table).

Progeny from the 64 BC_1_F_3_ plants selected were evaluated for physiological responses associated to drought tolerance and agronomical traits. Plants were grown in greenhouse, in Campina Grande, PB, during the dry season (Oct/2015-Feb/2016). BC1F_4_ plants seeds were sown in 30L pots containing sandy-loam textured soil previously limed and fertilized (NPK, 20:60:30, ammonium sulfate, single superphosphate and potassium chloride). Three cultivated genotypes were added to the assay: BR1 (Valencia-upright, tolerant to drought), Senegal 55–437 (Spanish-upright, tolerant to drought), and LViPE-06 (Virginia-runner, sensitive to drought) [3, 21, 22]. Plants were watered daily, maintaining field capacity, determined by gravimetric method after 72 h of draining [3]. At anthesis (24–25 days for upright cultivars and 34–35 days for runner LViPE-06) plants were submitted to 15 day of water restriction. Water replacement was based on crop evapotranspiration (ETC), estimated by an evaporation tank installed inside the greenhouse and the peanut crop coefficient [27]. The temperatures recorded during the assay, ranged between 18°C and 44°C. The relative humidity of the air was, on average, 68%.

An incomplete randomized block was adopted with 10 replicates. The following physiological traits were measured: stomatal conductance (*g*_s_, mol H_2_O m^-2^ s^-1^), transpiration rate (*E*, mmol H_2_O m^-2^ s^-1^), net photosynthetic rate (*P*_N,_ μmol m^-2^ s^-1^) and intercellular CO_2_ concentration (*C*_i_, μmol m^-2^ s^-1^). Based on these data, we estimated instantaneous carboxylation efficiency (*P*_N_/*C*_i_) and instantaneous water use efficiency (WUE, (μmol m^-2^ s^-1^)/(mmol H_2_O m^-2^ s^-1^)), as the ratio *P*_N_/*E* [28]. Data were collected from mid canopy fully expanded leaves, between 9:00 and 11:00 AM using an infrared gas analyzer (IRGA, ACD, LCPro SD, UK), coupled with light source at 1600 μmol m^-2^ s^-1^. Modulated chlorophyll fluorescence traits were estimated by Fluorometer OS5p (Opti-Sciences, Hudson, USA). Non-photochemical quenching (NPQ) was evaluated following methodology described in Kramer et al. [29].

Data were analyzed through uni and multivariate (non-hierarchical model) methods, using software GENES 2013.5.1 [30]. The UPGMA method was adopted as non-hierarchical model. A cophenetic correlation coefficient was estimated in order to adjust the model [31]. The Euclidean distance between the points representing the genotypes was used as a measure of relatedness [32].

### Validation of tolerant genotypes in field conditions

**Fig 2:**
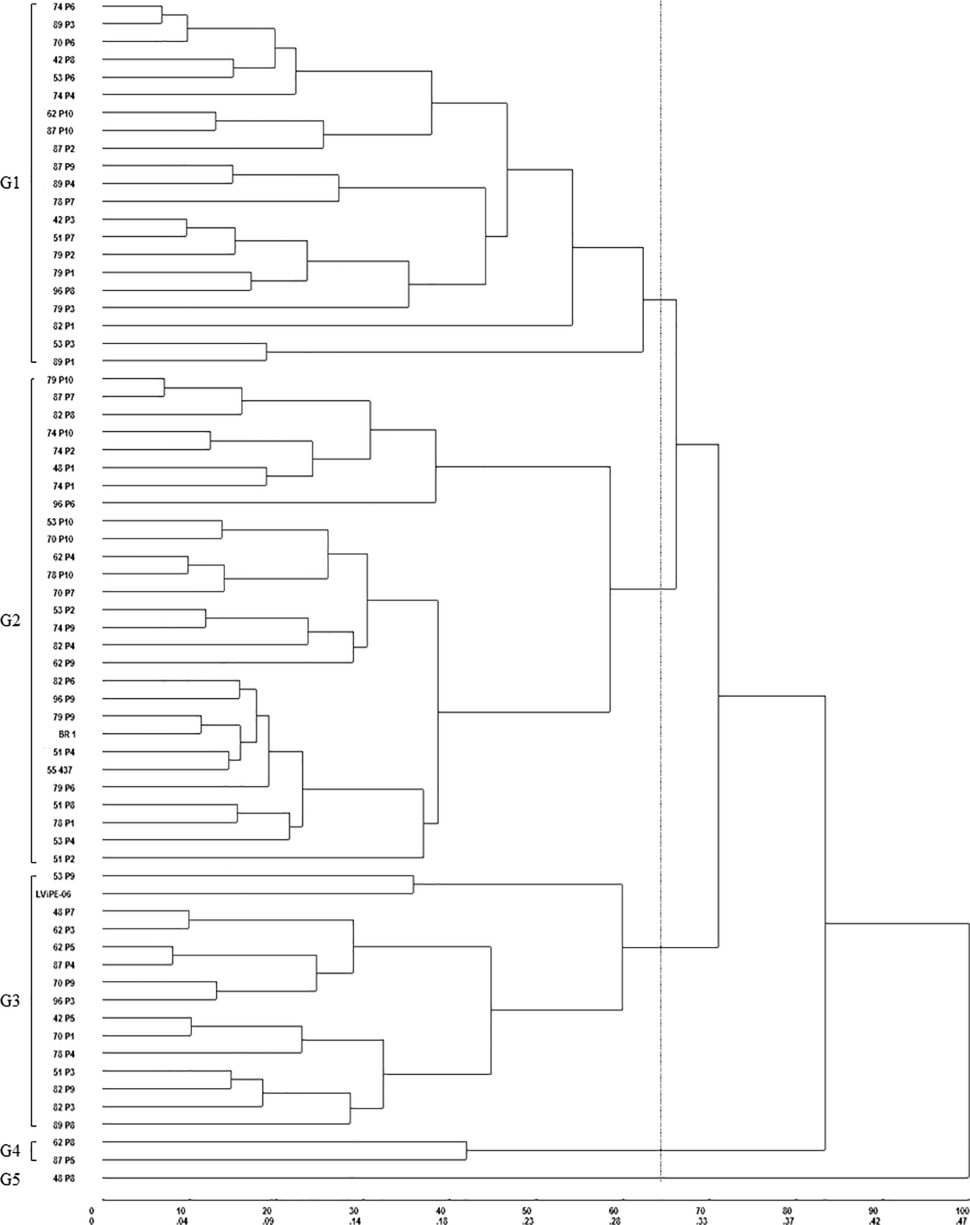
Dendogram obtained by UPGMA using 64 BC_1_F_4_ lines, based on physiological traits (*g*_s_, *E*, *P*_N_, *C*_i_, *P*_N_/*C*_i_, WUE, and NPQ). Coefficient of cophenetic correlation: 0.70 (*p* <0.01). Selection of groups based on genetic dissimilarity index (64.4%). G—Group.

Based on the dendrogram generated by UPGMA using physiological data (Fig 2), a 30%-selection was applied in plants clustered in same group of drought-tolerant cultivars (BR1 or Senegal 55–437). The seeds (BC_1_F_5_) of each plant were multiplied in Campina Grande, PB, under normal watering, between February and May 2016, adopting the same methodology as described before, for further use in validation assays.

BC_1_F_6_ lines were grown in the field, in a mid-sandy Entisol, in Lagoa Seca, PB (7°08'15.74''S, 35°50'20.05''W, 602 m, semiarid climate) during rainy season 2016 (May-Sep), and in a mid-sandy Vertisoil, in Campina Grande, PB, during rainy season (May-Aug, 2017). Soil of both places were previously limed (2t ha-^1^ dolomitic limestone) and fertilized (NPK, 20:60:30, ammonium sulfate, single superphosphate and potassium chloride). BR1 was used as control.

Each genotype was sown in one row (5 m length), spaced in 70 cm each. The density in each row was five plants/meter. A randomized complete block design was adopted with three replications. The crop management was followed according recommendations described in Santos et al. [26]. Data of first flowering was recorded at the beginning of reproductive phase of each line. At harvest, plants were maintained in the field for one week, for complete drying. Then, each genotype was evaluated for: number of pods per plant, number of seed per pod and pod yield (kg ha-^1^). Statistical analyses were done using software GENES, version 2013.5.1. [30]. *F* test was adopted to variance analysis. Means were compared by Tukey test.

## Results and discussion

### Initial selection procedures and clustering analysis

In this study, we aimed to produce breeding advanced lines, introgressing wild alleles from *A*.*duranensis* and *A*. *batizocoi* to improve peanut drought tolerance. An *A*. *batizocoi* x *A*. *duranensis* induced allotetraploid was crossed with a local elite drought tolerant cultivar, BR1. The F_2_ generation, obtained from this cross was backcrossed with BR1 and, from BC_1_F_2_ on, assays were carried out in green house and field in order to identify plants tolerant to drought. The rationale for using this approach was based mainly on *Arachis duranensis* being identified as a potentially good donor of alleles for drought tolerance. Leal-Bertioli et al. [1], carried out a study involving the effect of tetraploidization of wild *Arachis* on drought-related traits, and found an *A*. *duranensis* accession with conservative transpiration profile under water limited conditions. An induced allotetraploid was produced using this accession [11] and many anatomical and physiological traits were changed after tetraploidization [19]. However, the conservative transpiration profile was also present in the derived allotetraploid (data not shown). According to Brasileiro et al [33], transcriptome profiling of wild *Arachis* under water-limited environments, leaves and roots of *A*. *duranensis* revealed several transcripts involved with drought tolerance, such as Expansin, Nitrilase, NAC, and bZIP transcription factors. *A*. *duranensis* is a diploid wild annual species, native to low rainfall regions in Bolivia and Argentina, adapted to intermittent drought spells [1, 6] and in the present study, this trait was selected in the tetraploid backcrossed lines.

After backcrossing, 37 out of 87 BC_1_F_2_s had better harvest index out of which and 12 had better drought tolerance index than the recurrent parent, BR1 (S1 Table). After and three rounds of selection based on seed size, harvest index and drought tolerance index, 64 BC_1_F_4_ genotypes were planted in green house. They were submitted to 15 days of water stress and evaluated for physiological responses associated to drought tolerance and agronomical traits. During dry period, twelve genotypes, including LViPE-06, showed evident drought sensitivity (42 P5, 51 P3, 53 P9, 62 P3, 62 P5, 70 P1, 70 P9, 78 P4, 82 P9, 89 P8, and 96 P3), such as drastic loss of turgor and reduced growth, even after reestablishment of watering. The other remaining genotypes showed moderate behavior with only slight reduction of growth and leaf turgor.

**Fig 3:**
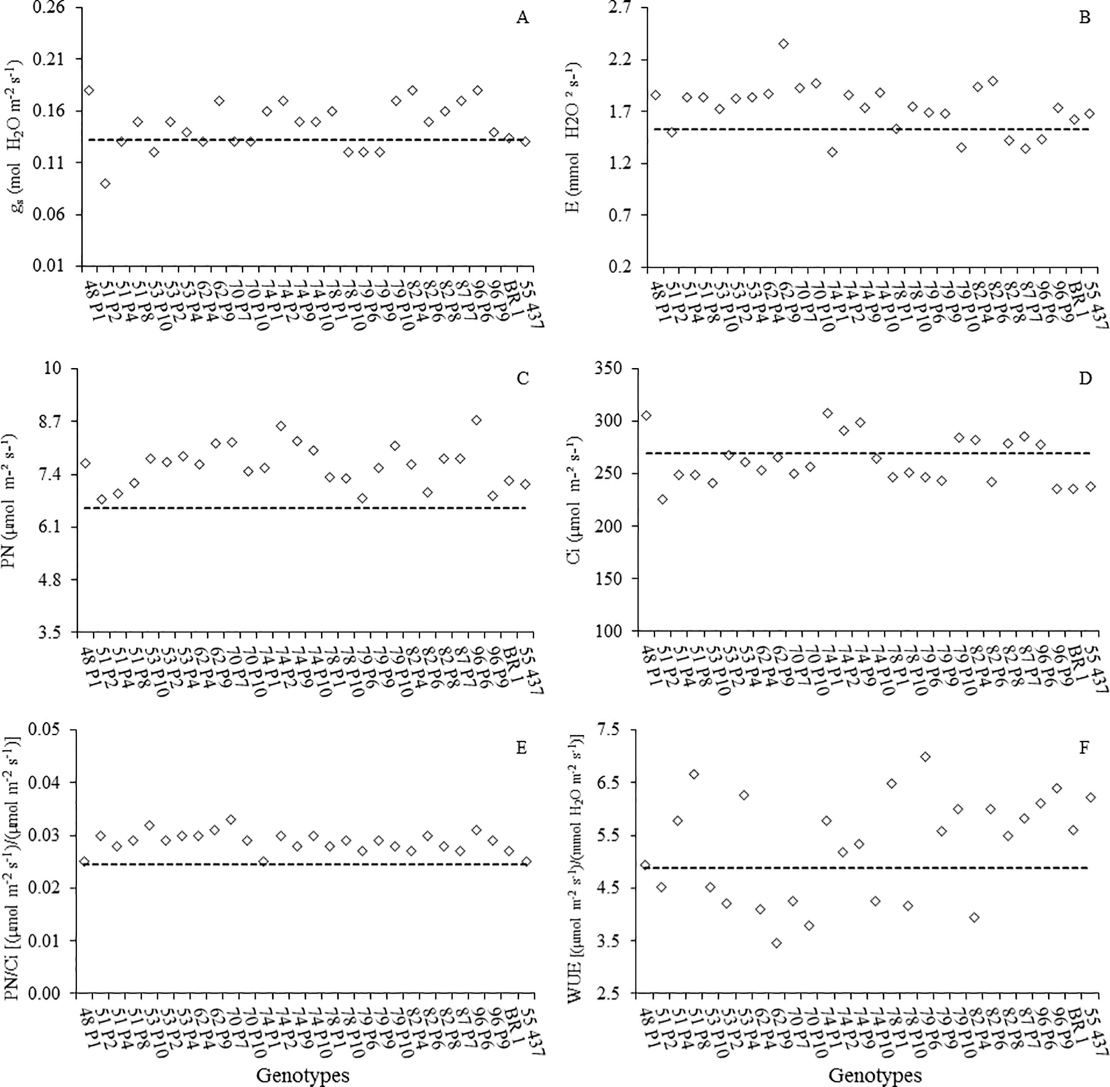
Gas exchange of peanut line clustered in G2 group. A- Stomatal conductance (*g*_s_), B- transpiration rate (*E*), C- net photosynthetic rate (*P*_N_), D- intercellular CO_2_ concentration (*C*_i_), E- instantaneous carboxylation efficiency (*P*_N_/*C*_i_), F- instantaneous water use efficiency (WUE). Dashed line is the estimated mean of 64 lines. BR1 and 55–437 (Controls).

All 64 genotypes were evaluated using seven drought-tolerance related physiological traits. The data were used to clustering analysis, based on UPGMA method. Five groups were found, among them the Group 2 was the most interesting because 26 genotypes clustered close to BR1 and Senegal 55–437 (Fig 2). Overall, these genotypes maintained high stomatal conductance (g)_s_ (Fig 3A), resulting in increased transpiration rate (*E*, Fig 3B). This combination favored the maintenance of net photosynthetic rate (*P*_N_, Fig 3C), reducing the intercellular CO_2_ concentration (*C*_i_, Fig 3D) in these plants, during the period of water restriction. As seen in Fig 3E, most genotypes showed instantaneous carboxylation efficiency (*P*_N_/*C*_i_) similar or higher than BR1. This indicates efficiency in CO_2_ fixation in low water availability. Eleven genotypes had superior water use efficiency than the control recurrent parent BR1 (Fig 3F). Additionally, eight out of 64 BC_1_F_4_ plants produced heavier pods and three produced heavier seeds (S1 Table). This indicates that that these genotypes were more tolerant to water stress, based on the experimental conditions adopted here.

**Fig 4:**
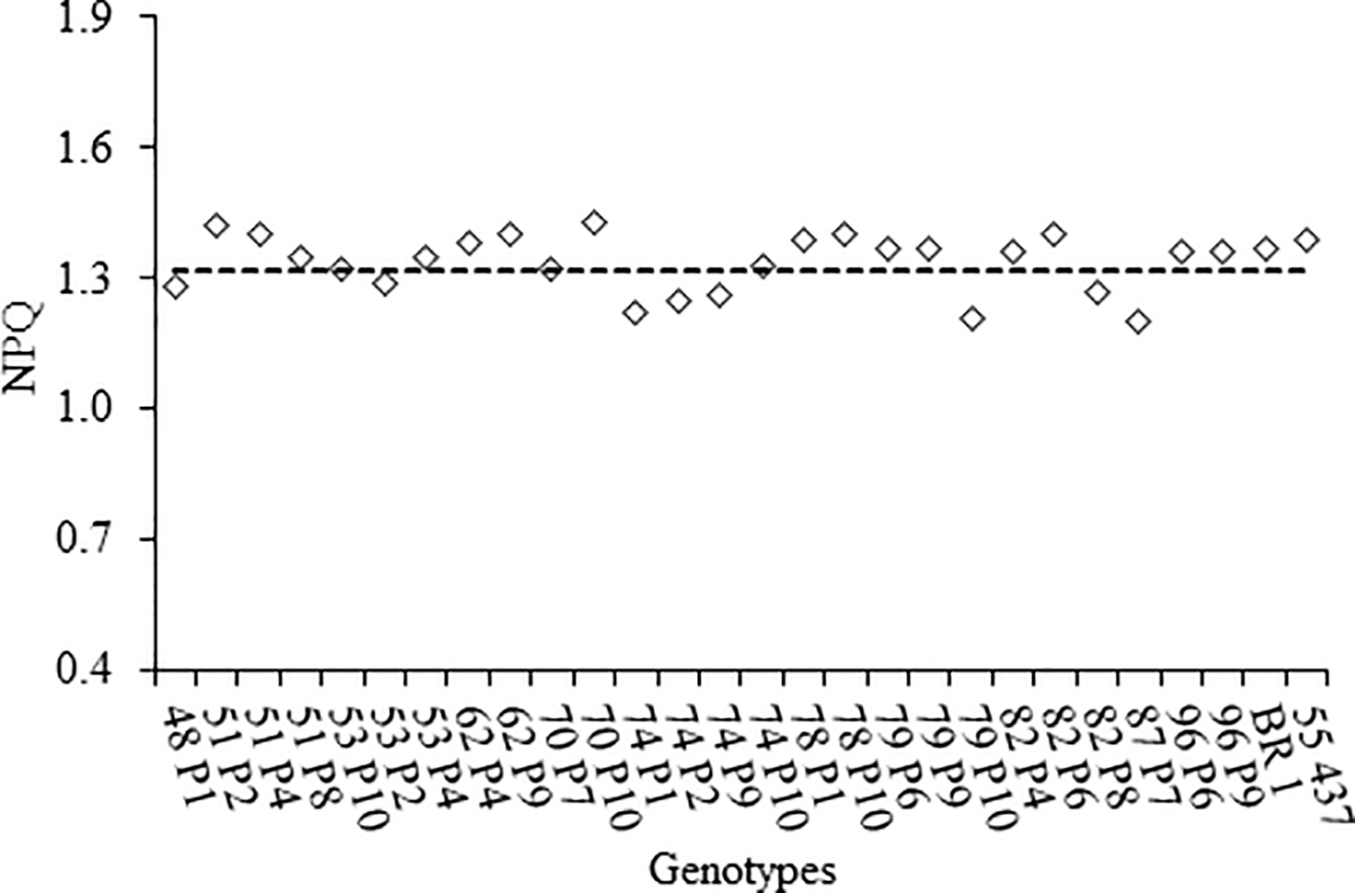
Non-photochemical quenching (NPQ) of peanut line clustered in G2 group. Dashed line is the estimated mean of 64 lines. BR1 and 55–437 (Controls).

Plants often regulate stomata closure under water deficit, reducing transpiration in order to overcome the stress period. This situation leads to reduction of CO_2_ influx. According to the literature, stomatal conductance (*g*_s_) is one of the main factors limiting photosynthesis in plants under water stress [2, 4]. As expected, stomatal conductance was positively correlated with net photosynthetic rate (Table 1). In semiarid environments, the occurrence of intermittent drought (also called *Indian summer* or *veranico*) during the rainy season is frequent and is usually associated with high solar radiation. This combination may lead to severe damage to the photosynthetic apparatus, and therefore, reduces substantially the CO_2_ fixation in plants. In order to avoid this damage, plants develop several protective mechanisms, such as non-photochemical quenching (NPQ), that is responsible for light energy balance with the photosynthesis [4]. In this study, the value of NPQ of 15 genotypes exceeded the general mean (Fig 4), among them, 10 were similar or higher than BR1, indicating that these genotypes were able to eliminate the excess energy, improving the functioning of the photosynthetic apparatus even under water stress. According to Kalariya et al. [4], that submitted several peanut genotypes to water restriction, intercellular CO_2_ concentration (*C*_i_), net photosynthetic rate (*P*_N_) and non-photochemical quenching (NPQ) are traits that provide wide variation in plants under water stress. The correlation of these traits is found in Table 1. Highly significant correlations were found to *gs* x *P*_*N*_ (0.57), *gs* x NPQ (-0.52), *gs* x *Ci* (0.76), *P*_*N*_ x *Ci* (0.62) and NPQ x *Ci* (-0.75), indicating that these can be used as surrogate traits for selection procedures in breeding programs for drought tolerance in peanut.

**Table 1:** Pearson correlation between physiological traits: Stomatal conductance (*g*_s_), net photosynthetic rate (*P*_N_), instantaneous carboxylation efficiency (*P*_N_/*C*_i_), instantaneous water use efficiency (WUE), non-photochemical quenching (NPQ), transpiration rate (*E*), and intercellular CO_2_ concentration (*C*_i_) of peanut lines.

Traits	*P*_*N*_	*P*_*N*_*/Ci*	WUE	NPQ	*E*	*Ci*
*gs*	0.57**	0.10^ns^	-0.38*	-0.52**	0.56**	0.76**
*P*_*N*_	-	0.61**	0.26^ns^	-0.50**	0.42*	0.62**
*PN/Ci*	-	-	-0.32^ns^	0.42*	0.39*	-0.47*
WUE	-	-	-	-0.18	-0.51**	-0.05^ns^
NPQ	-	-	-	-	0.49**	-0.75**
*E*						-0.19^ns^

^ns^ not significant

* and ** significant to *p*≤0.05 and *p*≤0.01, respectively.

**Fig 5:**
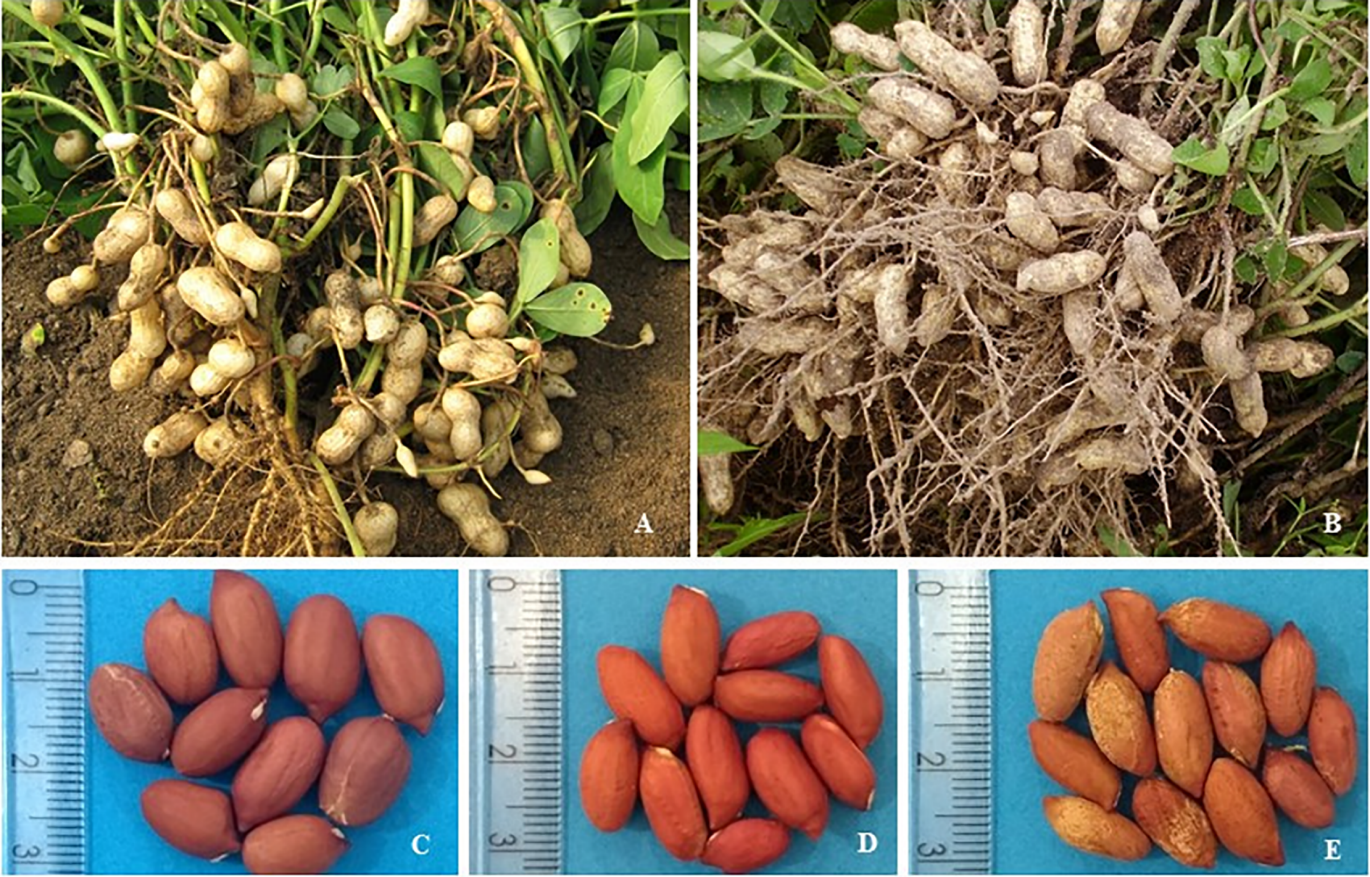
Phenotypical variants seen in pods and seeds of BC_1_F_4_ lines and BR1. A, D and E- Lines 53 P6, 48 P8 and 87 P2 showing small and constricted pods, and small and lighter seeds; B and C- Pods and seeds of BR1.

The BC_1_F_4_ lines evaluated, at the end of the cycle, presented pods with varied size and seed number, and seeds of smaller size than the recurrent parent, BR1 (Fig 5).

In order to identify the most promising lines in G2-group for further evaluation in field assay, a 30%-selection was applied, based on previous data of anthesis of 22–23 days after emergence, weight of pods/plant: ≥ 20 g, and number of pods/plant: ≥ 15. With these criteria, eight genotypes were selected for field evaluation.

### Validation of drought tolerant progenies through field assays

**Fig 6:**
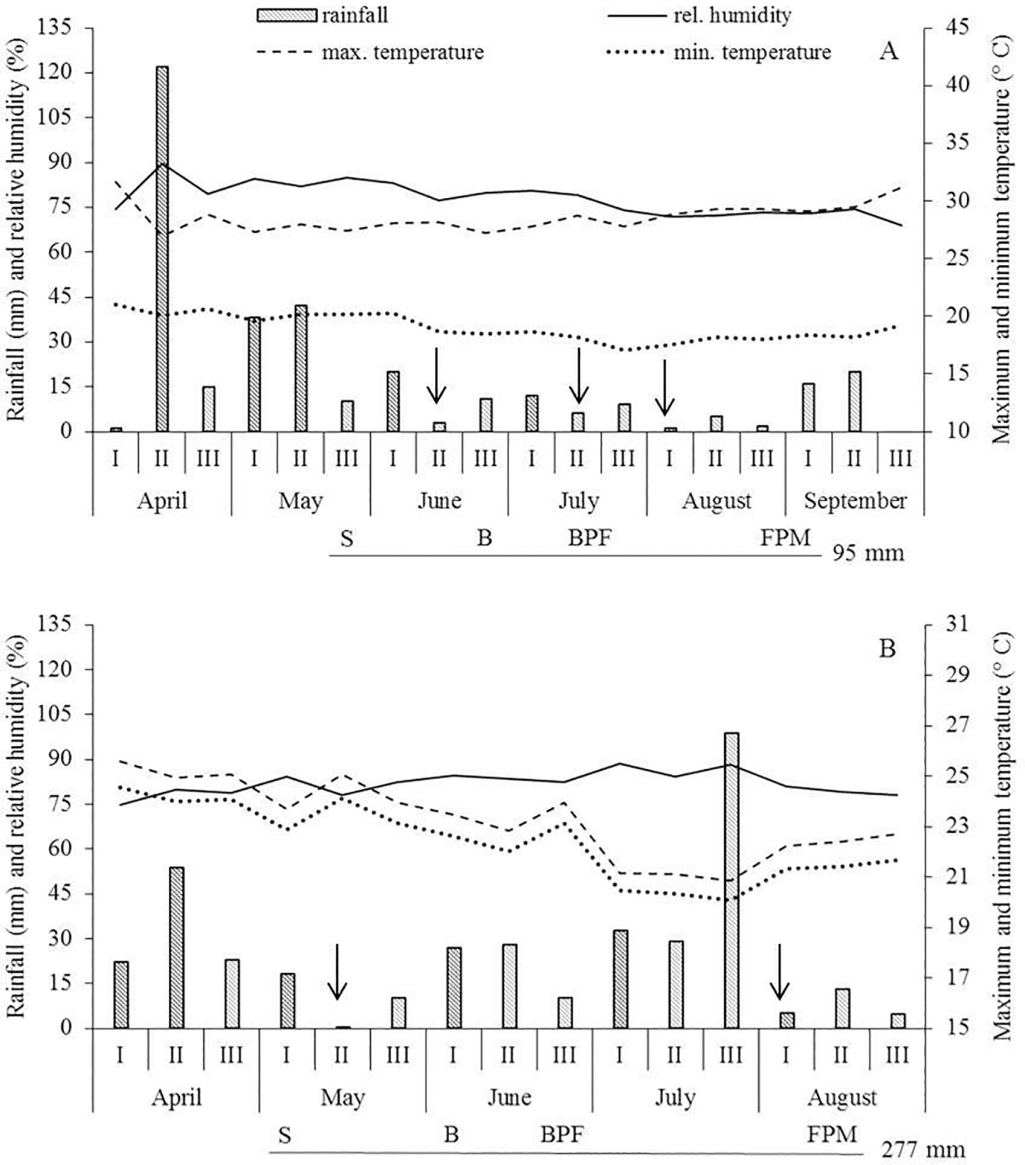
Climate data registered during assays in Lagoa Seca (A) and Campina Grande (B), PB, Brazil. S- sowing, B- blooming, BFP- beginning of pod formation, FPM- full pod maturation. Arrows mean periods of *Indian summer*.

The selected eight lines were tested for drought tolerance in two different environments, Lagoa Seca, PB and Campina Grande, PB, during the rainy period 2016 and 2017, respectively. The precipitation during the cycle was 95 mm and 277 mm, respectively. In Lagoa Seca, three periods of *Indian summer* lasted for nine to 16 days, at the critical times of blooming, beginning of pod formation and seed formation (Fig 6A). The low rainfall associated with high evaporation strongly influenced the production of lines. In Campina Grande, two 10-day periods of *Indian summer* were recorded at beginning of growth and at final of pod maturation (Fig 6B). Yield-related traits were measured in these two environments. The drought tolerant control and recurrent parent, BR1 showed high yield stability, with similar values in both environments (Table 2). Segregating lines, however, were widely influenced by environments (E), especially for number of pods per plant (NP/P) and yield (Table 2), both quantitatively inherited and dependent of crop management [34]. In Lagoa Seca (low rainfall), two lines, 53 P4 and 96 P9, had similar yield to BR1. In Campina Grande, in better conditions, these same lines plus 51P4 and 82P6 yielded more than BR1. This shows that under mild drought stress, wild introgressions, provided increased yield and under quite severe stress, yield is maintained at the same level as the recurrent parent.

**Table 2:** Summary of combined- variance analyses of anthesis (A), number of pods/plant (NP/P), number of seeds/pod (NS/P) and yield of introgression lines grown in Lagoa Seca (LS) and Campina Grande (CG), Paraíba, Brazil, during rainy season.

SV	DF	MS							
		A (dae)		NP/P		NS/P		Yield (kg ha-^1^)	
Bl/E	4	1.778		3.881		0.022		9889.575	
E	1	13.500^ns^		976.225**		1.176**		17794344.498**	
G	8	1.583^ns^		39.824^ns^		0.168^ns^		529735.225^ns^	
G x E	8	0.500^ns^		25.873**		0.141**		322708.331**	
Error	16	0.507		2.701		0.073		14856.925	
Mean		23.50		19.52		2.96		1608.29	
CV (%)	32	3.03		8.42		2.87		7.58	
		Means of traits in environments							
Genotypes		A (dae)		NP/P		NS/P		Yield (kg ha-^1^)	
		LS	CG	LS	CG	LS	CG	LS	CG
51 P4		22.7	23.3	15.6abB	25.7aA	2.6cBB	3.2aA	1149.8bB	2265.9abA
51 P8		23.0	24.0	14.3bcB	20.4bA	2.3dB	3.1aA	722.8cdB	2010.7cdeA
53 P4		23.3	23.7	17.7abB	28.3aA	3.0abA	3.1aA	1722.3aB	2487.5abA
78 P1		23.7	23.7	12.6cB	19.6bA	2.9bB	3.1aA	1044.3bcdB	1752.4eA
79 P6		23.0	24.7	15.9abB	27.5aA	2.9bB	3.1aA	1073.8bcB	1926.1deA
79 P9		23.3	24.7	13.3bcB	19.9bA	2.4dB	3.1aA	671.4dB	2173.6bcdA
82 P6		22.7	24.0	14.1bcB	26.8aA	3.0abA	3.0aA	878.9bcdB	2265.9bcA
96 P9		22.0	23.0	15.9abB	28.1aA	3.2aA	3.2aA	1807.8aB	2700.1aA
BR1		23.3	25.0	17.9aB	17.7bA	3.2aA	3.2aA	1841.0aA	1857.9deA

dae = days after emergence, SV- source of variance, DF- degree of freedom, MS- mean square, Bl- block, E- environment, G- genotype, CV- coefficient of variation, kg ha-^1^ - kilograms per hectare

^ns^- not significant

**- Significant, F test (*p*≤0.01).

Means with same letters do not differ statically. Letters in upper case represent comparisons between environment and lower case, among genotypes, by Tukey test (*p*≤0.05).

As to other traits, no G x E effect was found on number of days to anthesis (A) and number of seed per pod (NS/P). These results were expected since the selection of all eight lines in group G2 (Fig 2) were previously based on earliness and pod pattern of BR1.

The results obtained here are very significant because they reveal the genetic adaptation of the introgression lines. The genus *Arachis* is divided in nine sections and most of species are diploid with negligible fertility when crossed directly with *A*. *hypogaea* [6]. The genetic resources in the genus *Arachis* are extremely diverse, representing a valuable source of genes to environmental adaptation and tolerance to abiotic and biotic stresses [35]. In the literature, a few papers have reported the identification of diploid species with broad tolerance to water stress [1, 36, 37]. The inheritance of genes associated to drought is quite complex, and wild species harbor many agronomically unadapted traits. Therefore, backcrossing is necessary [1]. In an early generation of induced allotetraploid used in this work [BR1 x (BatDur)], the progenies showed phenotypic traits more similar to the wild species, especially traits associated to pods. However, a single cycle of backcrossing with BR1, together with selection, was enough to restore pod traits to a commercial standard.

**Fig 7:**
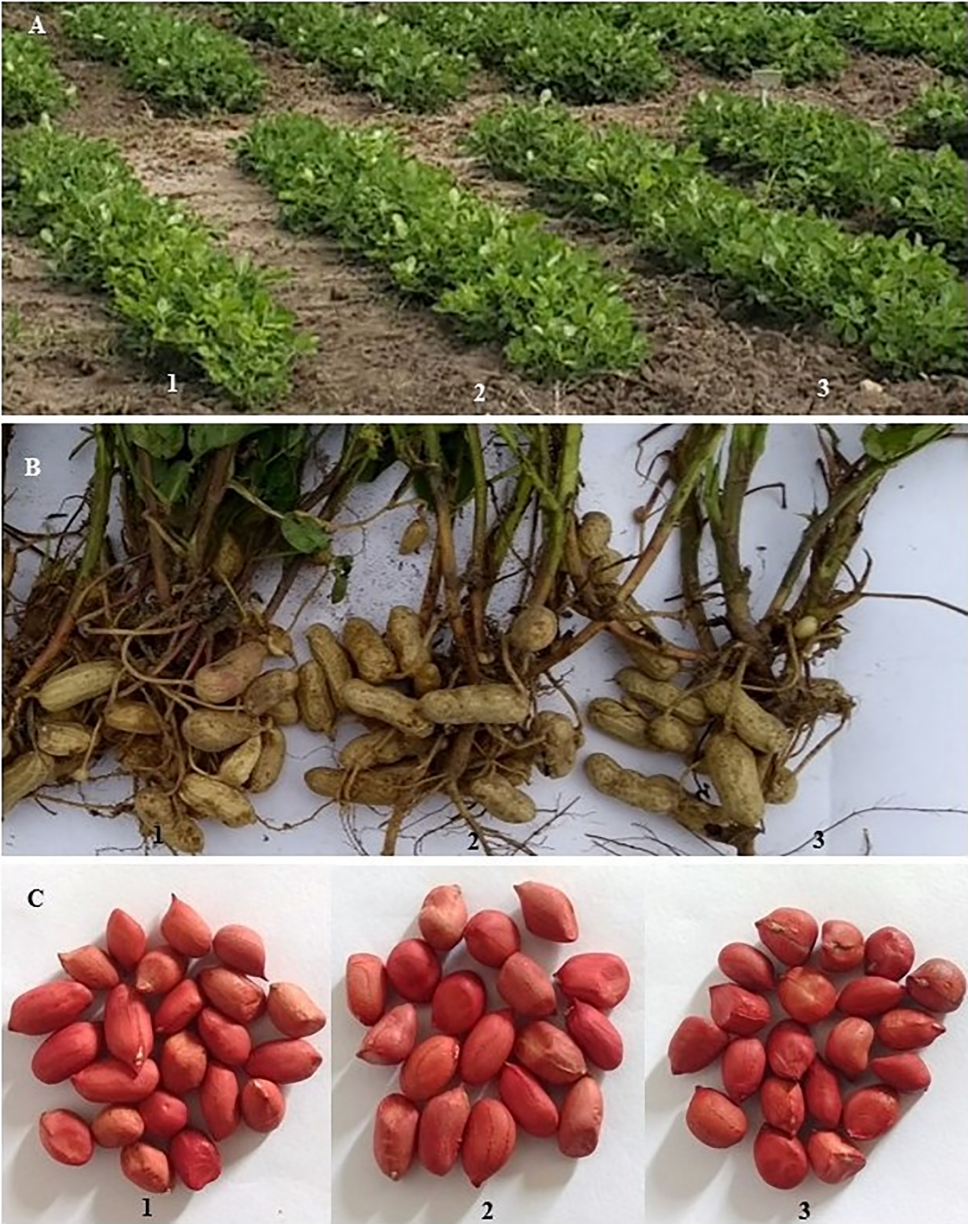
Detail of BC_1_F_6_ advanced lines grown in field. A- Plant canopy, B- Pod production, C- seed pattern of 53 P4 (1), 96 P9 (2), and *A*. *hypogaea* BR1 (3).

The two peanut lines found here, 53 P4 and 96 P9, are promising materials for drought tolerance-improvement because they were better than BR1 and some physiological traits of tolerance to drought were probably inherited from the wild species. The phenotypical profile of both lines are shown in Fig 7. The external aspects of these plants (height, canopy and pod conformation) were similar to BR1, indicating that in spite of the introgression of wild genes, the architecture of donor cultivar was recovered.

## Conclusions

The BR1 is a peanut cultivar widely grown in semiarid environment. It was commercially released in mid 90’s, and up to now, has broad acceptance by Northeastern farmers [20, 34]. The current Brazilian peanut cultivars were generated from *A*. *hypogaea* accessions, which has narrow genetic basis. The adoption of these new breeding lines represents an opportunity to broaden the genetic base of future cultivars, as well as to open the opportunity for the use of wild genetic resources in breeding programs, which are often maintained only in germplasm collections. The lines created here are very promising materials for advancement in peanut breeding for the semiarid environment.

The results presented here represent a great contribution to the peanut breeding developed to semiarid environment, especially since it deals with the valorization of wild species genes introduced for *A hypogaea*. Several germplasm banks in the world have thousands of accesses of species kept in order to maintain the integrity of the genetic heritage. The use, however, of such accesses for genetic improvement has been limited, due to methodological difficulties or chromosome barriers. In the present work, it is possible to recover the phenotypic pattern of BR 1, which is an earliness and high yield Valencia type. With the two lines found here, it is possible to advance the breeding works, with perspective of develop new cultivars, keeping the baggage of BR 1 and the inherited traits of the wild species. In addition, this work opens opportunities of new studies, involving the knowledge and interaction of new introgression genes in peanuts, especially for drought tolerance.

## Supporting information

S1 Table Data of four generations of lines derived from the induced allotetraploid (A. batizocoi x A duranensis)4x crossed and backcrossed with A. hypogaea subsp fastigiata cv BR1). (XLSX)

## Abbreviations

CO_2_: carbon dioxide
*g*_s_: stomatal conductance
E: transpiration rate
*P*_N_: net photosynthetic rate
*C*_i_: intercellular CO_2_ concentration
*P*_N_/*C*_i_: instantaneous carboxylation efficiency
WUE: instantaneous water use efficiency
NPQ: non-photochemical quenching
